# Urinary Incontinence and Quality of Life in Female Patients with Obesity

**DOI:** 10.1055/s-0038-1670626

**Published:** 2018-09

**Authors:** Christiana Campani Nygaard, Lucas Schreiner, Thiago Picolli Morsch, Rodrigo Petersen Saadi, Marina Faria Figueiredo, Alexandre Vontobel Padoin

**Affiliations:** 1 Graduate Program in Medicine and Health Sciences, Pontifícia Universidade Católica do Rio Grande do Sul, Porto Alegre, RS, Brazil

**Keywords:** obesity, urinary incontinence, bariatric surgery, obesidade, incontinência urinária, cirurgia bariátrica

## Abstract

**Objective** To analyze the prevalence of urinary incontinence (UI) in female patients with an indication for bariatric surgery, to investigate the potential risk factors and the impact on quality of life.

**Methods** A cross-sectional study with female patients with obesity. The evaluation consisted of a structured interview, a specific study form and quality of life questionnaires. The Poisson regression was performed to identify independent risk factors related to UI.

**Results** A total of 221 patients were enrolled; 118 of the study participants (53.4%) reported UI episodes. Mixed UI (MUI), stress UI (SUI) only, and urgency UI (UUI) only were reported by 52.5% (62), 33.9% (40) , and 13.5% (16) of these patients respectively. The prevalence of UI was increased by 47% among the women who had given birth vaginally and by 34% of the women who had entered menopause. Vaginal delivery and menopause were identified as independent risk factors related to UI. The mean International Consultation on Incontinence Questionnaire - Short Form (ICIQ-SF) score was 9.36 ± 4.9. The severity of symptoms was considered moderate in 53.3% (63) of the patients with UI.

**Conclusion** Urinary incontinence impacts quality of life negatively, and the prevalence of UI is high among obese patients. In the present study, vaginal delivery and menopause were independently associated with UI.

## Abstract (pt)

**Resumo:**

**Objetivo** Analisar a prevalência de incontinência urinária (IU), os fatores de risco e o impacto na qualidade de vida em pacientes femininas com indicação para realização de cirurgia bariátrica.

**Métodos** Estudo transversal com pacientes femininas obesas. A avaliação consistiu em entrevista estruturada, com questionários de estudo específico e de qualidade de vida. A regressão de Poisson foi utilizada para identificar os fatores de risco independentes para IU.

**Resultados** Um total de 221 pacientes foram incluídos; 118 participantes (53.4%) relataram episódios de IU. Incontinência urinária mista, IU de esforço e IU de urgência foram relatadas por 52.5% (62), 33.9% (40) e 13.5%(16) das pacientes, respectivamente. A prevalência de IU foi 47% maior em mulheres que tiveram parto vaginal, e 34% maior em mulheres que já entraram no período da menopausa. Parto vaginal e menopausa foram identificados como fatores de risco independentes para IU. A média da pontuação do International Consultation on Incontinence Questionnaire - Short Form (ICIQ-SF) foi de 9.36 ± 4.9. A severidade dos sintomas foi considerada moderada em 53.3% (63) das pacientes com IU.

**Conclusão** A IU impacta negativamente a qualidade de vida, e a prevalência de IU é maior em pacientes obesas. Neste estudo, parto vaginal e menopausa foram fatores de risco independentes para a ocorrência de IU.

## Introduction

Obesity is a chronic disease with high prevalence that is difficult to manage.^1^ It is a public health problem, and up to 1.9 billion people are affected by obesity or overweightness.^2^ The prevalence of obesity has doubled since the 1980s.^3^ By 2016, 39% and 13% of adults over 18 years of age were overweight and obese respectively.^2^ In the past, obesity was considered a problem found in developed countries; however, obesity and overweightness are currently drastically increasing in low- and middle-income countries, particularly in urban centers.^2^ ^3^

Obesity is responsible for several adverse health effects, including increased morbidities and greater mortality.^4^ Elevated risks are already observed for overweight individuals, and increase progressively with the development of obesity.^3^ Obesity causes functional disabilities, reduced quality of life and reduced life expectancy, and is known to contribute to increases in chronic diseases, including cerebrovascular and cardiovascular diseases, diabetes, sleep apnea and pelvic floor dysfunctions.^5^ ^6^ The negative effects of obesity on health include a strong association between obesity and urinary incontinence (UI).^7^

Obesity is the best established and most potentially modifiable risk factor in the development of UI.^8^ A positive association between UI and body mass index (BMI) has already been confirmed in certain studies.^7^ ^8^ There is a clear dose effect of weight on incontinence, with each 5-unit increase in BMI associated with a 20 to 70% increase in incontinence risk.^7^ Compared with normal-weight patients, obese patients have approximately twice the risk of presenting with UI.^9^ ^10^ Increased weight may aggravate or cause pelvic floor disorders by increasing both intra-abdominal pressure and chronic pressure on ligaments and nerves, leading to excessive stretching.^11^ ^12^

In addition, there are other recognized risk factors for UI. There is an increase in the prevalence of UI during the perimenopausal period.^9^ Most studies show a peak prevalence of UI, particularly stress UI (SUI), between the ages of 40 and 60.^13^ ^14^ Parity is another factor that is clearly associated with increased UI.^10^ ^15^ ^16^ ^17^ Cesarean sections appear to have a protective effect; nevertheless, UI often presents during pregnancy, and this manifestation is a predictive factor for postpartum UI regardless of the delivery method.^18^ ^19^ Fetal weight and advanced maternal age also appear to be risk factors for UI.^20^ A previous hysterectomy has been positively associated with the appearance of UI, although the only available data regarding this association are from observational studies.^21^

The aim of this study was to analyze the prevalence of UI in female patients with an indication for bariatric surgery and investigate potential risk factors.

## Methods

The present study was conducted in a reference center for morbid obesity. Ethical and research approvals were obtained from the applicable local committee.

An informed consent form was offered to patients who satisfied the study criteria. To the patients it was promised that all data would remain confidential, and that the obtained results would only be used for scientific purposes. The participants were recruited between June 2016 and September 2017. A total of 221 female patients aged over 18 years old who had been assessed for bariatric surgery were enrolled in the present study.

The initial evaluation was performed using a structured interview and a specific study form. Symptoms and quality of life were assessed based on the interview, and the results of the validated Portuguese version of the International Consultation on Incontinence Questionnaire - Short Form (ICIQ-SF) and Kings Health Questionnaire.^22^ The patients who reported episodes of UI once a week or less were considered symptomatic. The anthropometric data, bioimpedance results, and medical histories were obtained from the medical records.

Statistical analyses were performed using the Statistical Package for the Social Sciences (SPSS, IBM Corp., Armonk, NY, US) software, version 21. Descriptive statistics (mean, median, standard deviation, and range) were used to present numerical variable values. Numbers and absolute and relative percentage frequencies were used to present categorical variables. The Mann-Whitney U test was used to assess the statistical significance of differences between median values. Continuous data were analyzed using the Student *t*-test for related samples, and categorical variables were compared using chi-squared tests. Poisson regression was performed to identify independent risk factors related to UI. In the model, variables with *p*-values lower than 0.20 were included, and those with *p*-values lower than 0.10 were excluded. Multicollinear factors such as weight, excess weight, BMI, and waist circumference were incorporated into different models to improve the fit of the final model.

## Results

During the study period, a total of 325 patients were identified as eligible subjects; 221 of these patients consented to participate and completed the baseline assessment. The patients were divided into 2 groups (Group 1, patients with UI; Group 2, patients without UI). A total of 118 patients (53.4%) reported UI episodes.

The patients in Group 1 were significantly older than the patients in Group 2 (41.1 ± 12.1years versus 37.0 ± 9.1years, *p* = 0.006). Regarding the parameters used to evaluate the body composition of the patients, BMI, abdominal circumference and excess of weight were significantly higher in Group 1 than in Group 2. Menopause was more frequent in Group 1 than in Group 2 (23/116 (19.8%) subjects versus 8/102 (7.8%) subjects). These data are summarized in Table 1.

**Table 1:** Baseline demographic and clinical characteristics of the patients according to urinary incontinence status

Variables	Patients with UI (*n* = 118)	Patients without UI (*n* = 103)	*p-value*
Age (years)*	41.1 ± 12.1	37.0 ± 9.1	0.006
Weight (kg)*	118.7 ± 19.0	114.8 ± 17.5	0.121
BMI*	45.9 ± 7.6	44.0 ± 6.3	0.043
Waist circumference*	126.5 ± 13.1	122.8 ± 12.9	0.030
Waist-to-hip ratio*	0.93 ± 0.07	0.93 ± 0.19	0.704
Fat mass*	51.9 ± 3.5	51.5 ± 4.1	0.317
Excess weight (kg)*	53.8 ± 18.3	48.9 ± 16.0	0.041
Basal metabolic rate*	1583 ± 151	1565 ± 183	0.439
Hypertension	54/118 (45.8)	38/102 (37.3)	0.255
Diabetes	24/118 (20.3)	14/103 (13.6)	0.251
Asthma	11/118 (9.3)	7/103 (6.8)	0.661
Dyslipidemia	23/118 (19.5)	22/103 (21.4)	0.860
Smoking history	32/114 (28.1)	19/102 (18.6)	0.161
Hysterectomy	9/116 (7.8)	4/101 (4.0)	0.374
Menopause	23/116 (19.8)	8/102 (7.8)	0.020
Sexual activity	81/116 (69.8)	81/101 (80.2)	0.111
Parity			
0	31/116 (26.7)	39/103 (37.9)	0.208
1–3	75 (64.7)	57 (55.3)	
4–7	10 (8.6)	7(6.8)	
Mode of delivery			
Vaginal	30 (25.9)	17 (16.5)	0.088
Cesarean section	35 (30.2)	36 (35.0)	
None	31 (26.7)	39 (37.9)	
Both	20 (17.2)	11 (10.7)	

Abbreviations: BMI, body mass index; UI, urinary incontinence.

Note: *Data presented as mean ± standard deviation; *p-*value calculated with the chi-squared test. All other data presented as number/total of patients (%); *p-value* calculated with the chi-squared test.

A Poisson regression was performed to identify the independent risk factors related to UI (Table 2). After an adjustment, the following factors were associated with UI: vaginal delivery (*p* = 0.044) and menopause (*p* = 0.031). Among women who had given birth vaginally and women who had entered menopause, the prevalence of UI was increased by 47% and 34% respectively. Smoking, excess weight and having delivered both vaginally and via caesarean section exhibited borderline associations with UI after the adjustment.

**Table 2:** Multivariate analysis

Variables	PR (95%CI)	*p*-value
Smoking history	1.28 (0.98–1.66)	0.071
Mode of delivery		
None	1.00	
Cesarean section	1.22 (0.83–1.79)	0.301
Vaginal	1.47 (1.01–2.12)	0.044
Both	1.41 (0.94–2.10)	0.095
Menopause	1.34 (1.03–1.76)	0.031
Weight excess	1.00 (0.99–1.01)	0.073

Abbreviations: 95%CI, 95% confidence interval; PR, prevalence ratio.

With respect to the patients with UI (Group 1), 40 (33.9%), 16 (13.5%), 62 (52.5%), of these patients presented with SUI only, urgency UI (UUI) only, and mixed UI (MUI) respectively. The median urinary frequencies during the day and at night were 6 (5–8) voids and 2 (1–3) voids respectively, and 35/112 (31.2%), of the patients used pads due to UI. Coital incontinence was reported by 12/111 (10.4%). A total of 75/111 (67.6%) of the patients wanted a specific treatment for UI (Table 3).

**Table 3:** Data related to urinary incontinence

	Patients with UI (*N* = 118)
SUI only	40 (33.9)
UUI only	16 (13.5)
MUI	62 (52.5)
Coital UI	12/111 (10.4)
Frequency*	6.0 (5–8)
Nocturia*	2 (1–3)
Use of incontinence pads	35/112 (31.2)
Desire for a specific treatment	75/111 (67.6)

Abbreviations: MUI, mixed urinary incontinence; SUI, stress urinary incontinence; UI, urinary incontinence; UUI urgency urinary incontinence.

Note: *Median (range); other data presented as number/total of patients (%).

The results of the ICIQ-SF are described in Table 4. The mean ICIQ-SF score was 9.36 ± 4.9. The severity of symptoms was considered moderate in 53.3% (63) of the Group 1 patients. Most of these patients believed that they leaked small quantities: 79 (70.5%) of urine. The highest scores of the 9 domains of the Kings Health Questionnaire were in the domains “Impact of Urinary Incontinence,” “Measures of Gravity” and “General Perception of Health”, with mean scores and standard deviations of 44.3 ± 33.3, 41.2 ± 26.9, and 43.2 ± 21.5 respectively.

**Table 4:** ICIQ-SF scores

	Patients with UI (*N* = 118)
Total score *	9.36 ± 4.9
Severity	
Slight (1–5)	29 (24.5)
Moderate (6–12)	63 (53.3)
Severe (13–18)	19 (16.1)
Very severe(19–21)	7 (5.9)
Frequency of urine leakage	
Once a week or less	37 (31.5)
Twice to three times a week	31 (26.1)
Once a day	14 (11.8)
Several times a day	24 (20.3)
All the time	7 (5.9)
Amount of urine leaked	
Small	79 (70.5)
Moderate	26 (23.2)
Large	7 (6.3)
Leaks before patient can get to the toilet	70 (62.5)
Leaks when patient coughs or sneezes	85 (75.9)
Leaks when patient is asleep	20 (17.9)
Leaks when patient is exercising	31(27.7)
Leaks after patient urinates	34 (30.4)
Leaks for no obvious reason	23 (20.5)
Leaks all the time	4 (3.6)

Abbreviations: ICIQ-SF, International Consultation on Incontinence Questionnaire - Short Form; UI, urinary incontinence.

Note:*Total score = sum of questions 1, 2 and 3; other data presented as number/total of patients (%).

## Discussion

Urinary incontinence impacts quality of life negatively, and the prevalence of UI is high in patients with obesity. In the present study, UI affected more than half (53.4% (118)) of the patients with obesity; this finding was in agreement with previously reported results.^23^

Urinary incontinence was more prevalent in patients after menopause. The patients in Group 1 were older than those in Group 2, and this was statistically significant, although the difference was not a marked one. Since the tissues involved in the female urinary continence mechanism are sensitive to estrogen, estrogen deficiency after menopause may be an etiological factor in the development or progression of UI.^24^ This effect did not appear to be attenuated by the peripheral conversion of estrogen, which is particularly common in patients with obesity.^25^

Vaginal delivery was the other independent factor associated with a higher prevalence of UI. As it has been observed in other studies, this effect was most evident in the third and fourth decades of life, but was attenuated in middle age and absent for elderly subjects.^13^ ^26^ ^27^ Thus, the aforementioned finding could be explained by the fact that the mean ages of the examined subjects were 41.1 ± 12.1 years versus 37.0 ± 9.1 years for Group 1 and Group 2 respectively.

Urinary incontinence was more common in patients with higher BMIs, excess of weight and waist circumference. In most studies, a strong association is observed between increasing weight and UI. Each 5-unit increase in BMI was associated with ∼ 20 to 70% increase in the risk of daily incontinence.^7^ However, in our sample, in which all of the patients had obesity, none of those parameters were independently related to UI.

The most prevalent type of UI observed in the present study was MUI, followed by SUI. The association between UI and obesity is known to be stronger for SUI and MUI and more modest for UUI.^9^ ^28^ ^29^ ^30^ Cohort studies have also suggested that an earlier appearance of obesity is associated with a higher probability of developing UI in middle age.^31^ There is evidence that obesity increases the incidence of SUI, but increases in metabolic syndrome are more strongly associated with IUU.^32^ Increased weight may aggravate or cause pelvic floor disorders by increasing intra-abdominal pressure and chronic pressure on ligaments and nerves, leading to excessive stretching.^11^ ^12^

This study benefits from the use of a validated incontinence questionnaires, which enable the differentiation of incontinence type, frequency, and severity based on the self-reports of the patients. The use of this type of questionnaire allows for better interpretation of the impact that patients' symptoms have on their quality of life.

Another strength of our study is the number of obese patients who were enrolled. In many studies, an obese population is compared with a normal BMI population. Our data are from patients with a mean BMI of class III obesity.

An important limitation is the lack of an objective outcome to compare with subjective measures. Our data could be stronger had we performed a pad test and/or an urodynamic assessment.

Obesity is known to be associated with many medical issues. Recently, the association between obesity and UI, which impacts quality of life, has been widely researched. The impact of obesity on pelvic floor function and consequently UI cannot be underestimated. This effect is particularly relevant at the present time, given the increasing prevalence of obesity and, therefore, UI. Urinary incontinence is more than twice as prevalent as diabetes and dyslipidemia, and is more prevalent than hypertension. In this study, most of the patients with UI stated that they wanted a specific treatment for their symptoms; such treatment needs to be offered by health professionals.

## Conclusion

In our sample of patients with obesity, UI was highly prevalent and greatly impacted quality of life. In the present study, vaginal delivery and menopause were independently associated with a higher prevalence of UI.
